# Optimization of Micropump Performance Utilizing a Single Membrane with an Active Check Valve

**DOI:** 10.3390/mi9010001

**Published:** 2017-12-21

**Authors:** Gia Thinh Bui, Jung-Hao Wang, Jr-Lung Lin

**Affiliations:** 1 Department of Electrical and Mechanical Engineering, Hai Phong University, 171 Phan Dang Luu Road, Kien An District, Hai Phong City 180000, Vietnam; buigiathinh1784@gmail.com; 2 Material and Chemistry Research Laboratories, Industrial Technology Research Institute, Hsinchu 31040, Taiwan; wang.rainleo@gmail.com; 3 Department of Mechanical and Automation Engineering, I-Shou University, Kaohsiung City 84001, Taiwan

**Keywords:** micropump, active check-valve, bottom-protruding structure, pumping efficiency

## Abstract

In this study, we successfully designed and tested a new micropump that utilizes an active check valve and bottom-protruding structure to achieve sample transportation. We performed theoretical analyses and numerical simulations to determine the optimal location of the active check valve. We also experimentally analyzed variations in the generated flow rate with respect to the pneumatic frequencies, actuated air pressures, and locations of the active check valve. The experimental results indicate the optimum air pressure, driving frequency, and location of the active check valve to be 68.9 kPa, 26.0 Hz, and 2.0 mm, respectively. We obtained a maximum pumping rate of 488 μL/min and a maximum pumping efficiency of 35.4%. The proposed micropump could perform a crucial function in the transportation of microfluids and could be incorporated into micro total analysis systems.

## 1. Introduction

In recent decades, microfluidic chips have been successfully introduced to provide a means for biochemical analysis, drug delivery, toxic test, and nucleic acid synthesis. The benefits of microfluidic applications include the incorporation of biomedical sample delivery, testing, and analysis, and, accordingly, dramatically reducing the manual labor required to manually participate in labor multi-step sample handling and processing, as well as progressing data quality and quantitative analysis. Microfluidic applications also reduce the overall cost and time of measurement, while improving sensitivity and the specificity of analysis. Additionally, microfluidic components, which include microchannels, microvalves, micropumps, micromixers, microsenors, and microreactors, have been successfully incorporated into the microfluidic platform. Of these components, micropumps are the most important because they perform crucial functions in sample delivery and manipulation in micro total analysis systems. Designing an efficient micropump will be a prospective challenge in miniaturized biomedical systems.

Micropumps can be briefly classified as either non-mechanical- or mechanical-actuator dependent, indicating whether their parts are fixed or movable, respectively. Non-mechanical pumps transfer momentum to fluid by converting another form of non-mechanical energy into kinetic energy, for which a number of electrokinetic [1], electroosmotic [2], acoustic streaming [3], magnetohydrodynamic [4], capillary [5], and electrowetting [6] principles are reported in the literature. On the contrary, mechanical pumps require an actuation diaphragm to provide a volume stroke. Typically, mechanical actuation conveys fluids by coupling the deformation of a flexible membrane to changes in pressure difference of fluid. Actuation mechanisms are driven on electrostatic [7,8,9], electromagnetic [10,11], pneumatic [12,13,14], thermopneumatic [15,16], or piezoelectric [17,18,19] principles. Traditionally, actuation diaphragms are classified as involving either a reciprocating displacement pump or a peristaltic pump. A reciprocating displacement pump is obtained using multiple layers and functions as an actuated diaphragm with two passive check valves that help fluids to charge or discharge. These two passive check valves are complicated to fabricate as well as easily fatigued and thus prone to damage. Moreover, they are difficult to precisely control. Alternatively, micropumps used to actuate by three membranes that operate in a series are sometimes categorized as peristaltic micropumps [20,21,22,23,24]. These have been confirmed to be effective in conveying fluids in microchannels and, accordingly, are readily integrated with a bio-chip. Peristaltic pumps use three actuation membranes to selectively compress and clog, which enables the motion of the fluids. Each actuation diaphragm operates to displace volume for forward pumping and also serves as a check valve to block the flow. Therefore, the actuation diaphragms exhibit both volume displacement and check-valve functions. One advantage of the peristaltic micropump is that the direction of flow is dependent on the sequence of diaphragm actuations. As such, peristaltic micropumps are bidirectional pumps. Micropumps use various vertical [20,21,22,23,24] or horizontal [25] actuation mechanisms, which increase their structural complexity and the manufacturing required for multiple-layer alignment and assembly. The objective of this study was to design and reap the benefits of both a reciprocating displacement pump and a peristaltic pump. We designed an easily fabricated and low-cost micropump for achieving high throughput volumetric flow rates. The advantages of the proposed micropump are a single reciprocating membrane with an active check valve incorporated into a bottom-protruding structure, instead of the traditional two passive check valves. This micropump generates a peristaltic-like actuation effect. Furthermore, the proposed micropump could be integrated with perfusion three-dimensional (3D) articular chondrocyte cell culture systems [26].

In this study, we designed a new micropump for transporting media that utilizes a single pneumatically actuated membrane with an active check valve and a bottom-protruding structure. We used both theoretical and numerical models to determine the optimal location of the active check valve on the proposed micropump. Moreover, we used the design parameters of applied air pressure and operating frequency to experimentally investigate the transporting effect.

## 2. Theoretical and Numerical Methods

The larger deformation of the pneumatically actuation membrane can be approximately expressed as shown as below [25]:

(1) $$\frac{w_{o}}{a}={\left(\frac{4}{\pi }\right)}^{2}{\left[\frac{(1-\nu^{2})\left(\beta \frac{b}{a}+\gamma \lambda \right)}{4\cdot \left[\frac{\alpha b}{a}+\left(9-\alpha \right)\lambda +\frac{2}{\lambda }+\frac{9}{\lambda^{3}}\right]}\frac{Pa}{Eh}\right]}^{\frac{1}{3}}$$

Here, *P* and *h* are applied pressure and membrane thickness, respectively. $\alpha =\frac{128}{5\pi }$, $\beta =\frac{\pi^{2}}{4}$, $\gamma =2-\frac{\pi^{2}}{4}$, and $\lambda =\frac{\left(b-\bar{c}\right)}{a}=2.63$. *w_o_* is the z-directional maximum displacement. *a* and *b* are the short-side (longitudinal) and long-side (transversal) lengths, respectively. Equation (1) can be theoretically calculated to obtain the maximum deformation of the rectangular membrane for the different pneumatic pressures.

The profile of the z-directional displacement is expressed as follows [25]:

(2) $$w=\{\begin{array}{cc} w_{0}\cos\frac{\pi y}{a} & 0\leq x\leq \bar{c}/2\\ w_{0}\cos\frac{\pi y}{a}\cos\frac{\pi (x-\bar{c}/2)}{\left(b-\bar{c}\right)} & \bar{c}/2\leq x\leq b/2 \end{array}$$

Here, *c* is a curve-shaped length expressed by $c=\bar{c}\cos\frac{\pi y}{a}$. $\bar{c}$ is a plate-shaped segment at *y* = 0.

To evaluate the pumping rate of the proposed micropump, we optimized the design using a flexible membrane pneumatically activated by top air pressure. We performed a numerical simulation to investigate the deformation of the Polydimethylsiloxane (PDMS) membrane and to design the location of the active check valve. We numerically simulated the deformation mechanism using a commercial software (CFD-ACE+, CFD-RC, Huntsville, AL, USA). We simulated the deformation mechanism using the stress as well as grid deformation modules. We conducted the auto-remesh function to ensure smooth motion in the deformation process of the moving boundary. We activated the activate stress to simulate the volume displacement of membrane. The density (*ρ*), Young’s modulus (*E*), and Poisson ratio (*ν*) of the PDMS membrane are assumed to be 970 kg/m^3^, 1.4 MPa, and 0.5, respectively [25]. The numerical dimension of the pneumatically actuated membrane is 7.5 × 2.5 × 0.3 mm^3^. In this simulation, a 3D numerical domain was discretized into approximately 800,000 cells with structured hexahedral meshes. We used a stringent residuals criterion (less than 10^−8^) and a nonlinear stress residuals criterion (less than 10^−4^) between each iterative solution step to ensure solution convergence.

## 3. Materials and Methods

### 3.1. Design of the Micropump Chip

**Figure 1:**
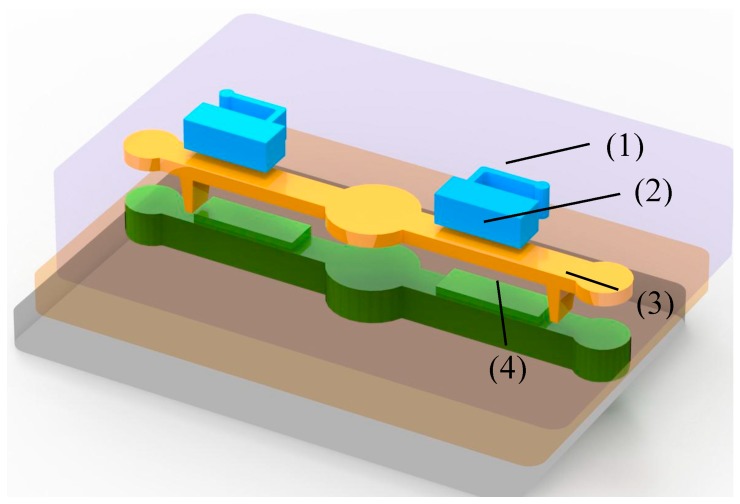
Schematic illustration of the proposed micropump. (**1**), (**2**), (**3**), and (**4**) indicate the air chamber, actuated membrane, active check valve, and bottom-protruding structure, respectively.

The proposed pump consists of three PDMS-based layers. Figure 1 shows the design of the proposed micropump, including the air chamber, actuation membrane, active check valve, and bottom-protruding structure. The dimensions of the actuated membrane are 7.5 (length) × 0.3 (thickness) × 2.5 (width) mm. The dimensions of the active check valve are 0.95 × 0.5 × 1.95 mm. The connected air channel is designed to be 0.1 mm wide. The dimension of fluidic channel is 17.5 mm in length, 2.0 mm in width, and 1.0 mm in depth. And the locations of the active check are 1.6 mm, 1.8 mm, and 2.0 mm, respectively.

First, we fabricated master molds of the microstructures on polymethylmethacrylate (PMMA) plates by utilizing a computer numerical control (CNC) machine (Roland Inc., EGX-400, Osaka, Japan) equipped with a 0.5-mm drill bit, with a rotating rate and feed rate of 27,000 rpm and 15 mm/min, respectively. These higher rotating and lower feed rates yielded a smooth surface on the PMMA plates. Then, we performed a PDMS casting process to produce inverse-image molds of the air chamber, actuation membrane with an active check valve, and microchannel with a bottom-protruding structure. Finally, we bonded these three-layer PDMS structures together utilizing an oxygen plasma treatment to produce the completed micropump chip.

### 3.2. Working Principle

**Figure 2:**
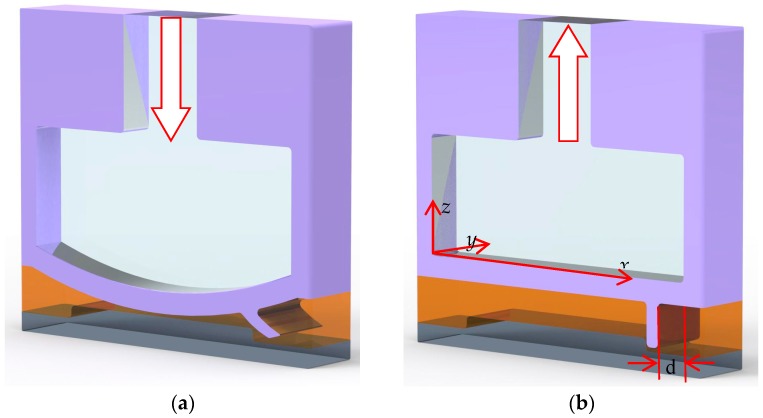
Schematic illustration of the actuation mechanism of the micropump. (**a**) The actuated membrane is activated by an active check valve to generate forward flow. (**b**) The active check valve is blocked by the bottom-protruding structure to prevent backflow. Red arrows indicate air flow in/out.

The proposed micropump consists of four parts: an actuated membrane, an active check valve, a bottom-protruding structure, and a microfluidic channel, as shown in Figure 2. In Figure 2a, the actuated membrane is activated by compressed air pressure, which lifts the active check valve in response to the deflection of the actuated membrane to generate the forward flow of the fluid. Conversely, as air is expelled from the air chamber, the active check valve will recover its original position, i.e., blocked by the bottom-protruding substrate to avoid to backflow, as shown in Figure 2b. The time-phased displacement of successively actuated membranes, which is caused by the externally compressed air through the pneumatic tube, generates a reciprocating activation effect that accelerates the fluid along the microchannel. As such, liquids can be smoothly transported by a series of operations in the proposed micropump. The location distance (d) is defined from the front rim of the air chambers to the position of the active check valve, as shown in Figure 2b.

### 3.3. Experimental Setup

The experimental system involved an air compressor (JUN-AIR Inc., mdr2-1a/11, Kawasaki-shi, Japan), a functional control circuit, and two electromagnetic valves (EMVs) (SMC Inc., s070 m-5bg-32, Taoyuan City, Taiwan) to evaluate the performance of the proposed pump. We conducted a series of tests to measure the pumping rates associated with the operating frequency, pneumatic pressure, and location of the active check valve. During the experimental observations, we positioned the proposed micropump under an optical digital camera (Olympus, E-5P, Tokyo, Japan) with an image-capturing system (Photometrics, CoolSNAP HQ2, Tucson, AZ, USA ) to acquire the motion of the fluid.

## 4. Results

### 4.1. Estimation of the Membrane Deformation

**Figure 3:**
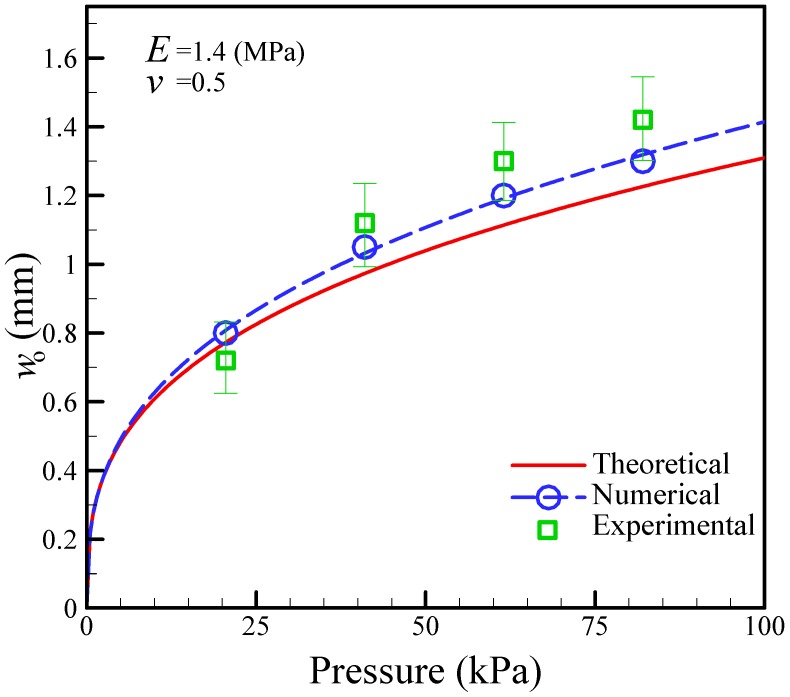
Comparison of maximum deformations between theoretical calculations, numerical simulations, and experimental measurements at different air pressures.

Figure 3 shows the relationship between the maximum deformation and the pneumatic pressure, as determined by theoretical analysis, numerical simulation, and experimental measurement. The numerical simulations are in reasonable agreement with the experimental measurements in comparison to the theoretical calculations. Although the numerical results better predict the deformation of the actuated membrane with the active check valve, the finite element method (FEM) employed requires a lot of time to calculate the deformation of the membrane. The theoretical analysis enables the easy prediction of the mechanism behaviors of the membrane. From these results, the maximum deformation of the actuated membrane obtained was 1.0 mm for an air pressure of 68.9 kPa. In this study, we designed the operating pressure to be 68.9 kPa since the depth of the microchannel is 1.0 mm.

**Figure 4:**
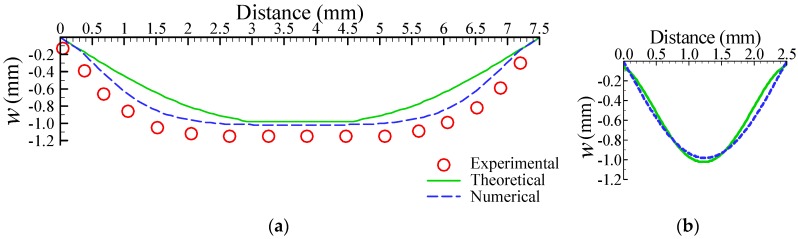
Theoretical, numerical , and experimental results for the (**a**) x-directional and (**b**) y-directional deformations at a pneumatic pressure of 68.9 kPa.

We theoretically, numerically, and experimentally investigated the deformation of the actuated membrane structure under a pneumatic pressure of 68.9 kPa. Figure 4a,b show numerical representations of the deformation profiles of the x-directional (see Figure 2a) and y-directional distance (see Figure 2b), respectively. The numerical deformation profiles of the x-direction also display two segments: plate- and curve-shaped profiles. In contrast, the deformation of the y-direction exhibits a symmetric curve-shaped profile. These deformation characterizations of the rectangle membrane are the same as those of an earlier study [25]. Based on the theoretical, numerical, and experimental results, we located the position of active check valve in a range of 0–2.5 mm or 5.0–7.5 mm, i.e., the distance from the rim of the actuated membrane is 0–2.5 mm. In this study, we fabricated three location distances of the active check valve for our experimental investigations, at 1.6 mm, 1.8 mm, and 2.0 mm, respectively.

**Figure 5:**
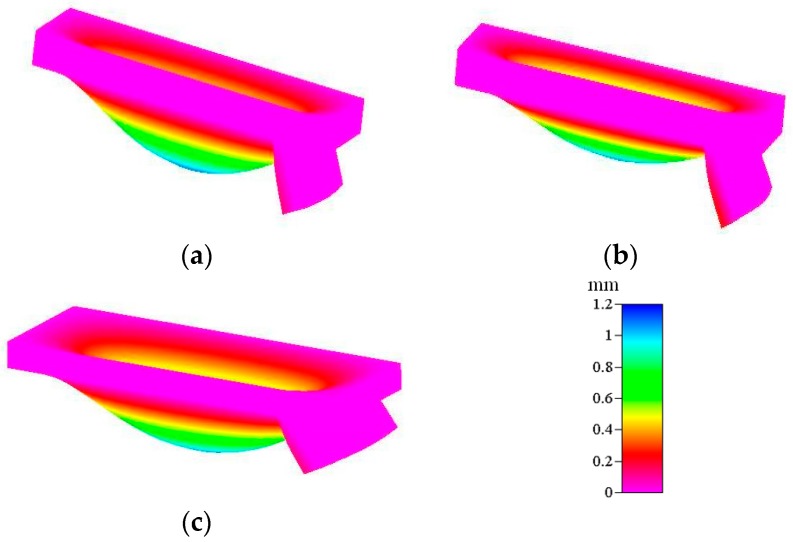
Deflections of the actuated membrane with the active check valve at different locations: (**a)** 1.6 mm; (**b**) 1.8 mm, and (**c**) 2.0 mm at a pneumatic pressure of 68.9 kPa.

The experimentally obtained motion of the active check valve in response to the actuated membrane is quite complicated, especially in the presence of actuated membrane deformation. Therefore, numerical simulation is important in the evaluation of the motion of the actuated membrane with respect to the active check valve. Figure 5 shows the lifting of the active check valve by the actuated membrane for the 1.6 mm, 1.8 mm, and 2.0 mm locations at an external pressure of 68.9 kPa. We numerically obtained a maximum membrane deflection of 1.04 mm at an applied air pressure of 68.9 kPa. This numerical calculation is in reasonable agreement with the later experimental observations. The pumping effect of the proposed micropump is dependent on the lifting deflection of the active check valve. In the design principle of the proposed micropump, a higher lifting deflection yields a higher pumping rate, which results in a lower flow resistance. Conversely, a lower lifting deflection yields a lower pumping rate due to the higher flow resistance. The higher lifting deflection of the active check valve is shown in Figure 5c, i.e., at the location of 2.0 mm from the rim of the actuated membrane.

### 4.2. Locations of the Active Check Valve

**Figure 6:**
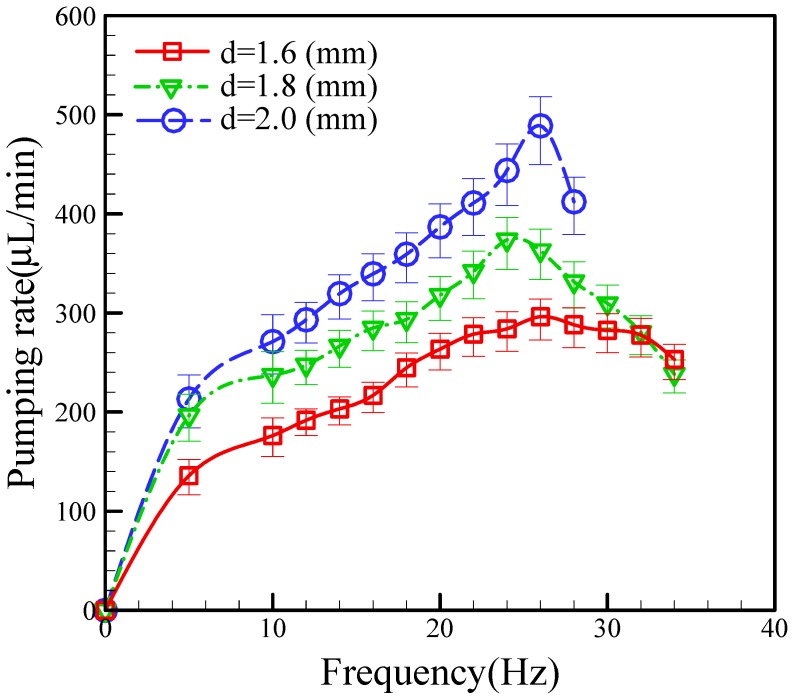
Relationship between pumping rate and different locations of the active check valve as a function of operational frequency at a pneumatic pressure of 68.9 kPa.

The contribution of this study is our design of a new pneumatic micropump that uses an actuated membrane with an active check valve and a bottom-protruding structure, which still provides a reasonable pumping rate even at a smaller scale. Using an active check valve makes this fluid-driven system more compact than previous peristaltic micropumps. Many factors affect the pumping rate of the micropump, including the driving frequency, applied pressure, and location of the active check valve. We found the effect of the different locations of the active check valve to mainly affect the pumping rate. We tested three distances from the active check valve to the rim of actuated membrane (1.6 mm, 1.8 mm, and 2.0 mm) at a constant pneumatic pressure of 68.9 kPa. Figure 6 shows a plot of the relationship between the pumping rate and the different locations of the active check valve as a function of operational frequency, in which we can see that the pumping rates for the different locations increase with increasing the frequency. The pumping rates also increase with increasing the distance of the active check valve. Specifically, the maximum pumping rates achieved for the check valve distances of 1.6, 1.8, and 2.0 mm were 296 μL/mm, 373 μL/mm, and 488 μL/mm, respectively. We found the optimum operational frequencies for the channels with check valve distances of 1.6, 1.8, and 2.0 mm to be 26.0 Hz, 24.0 Hz, and 26.0 Hz, respectively.

### 4.3. Pumping Parameters

**Figure 7:**
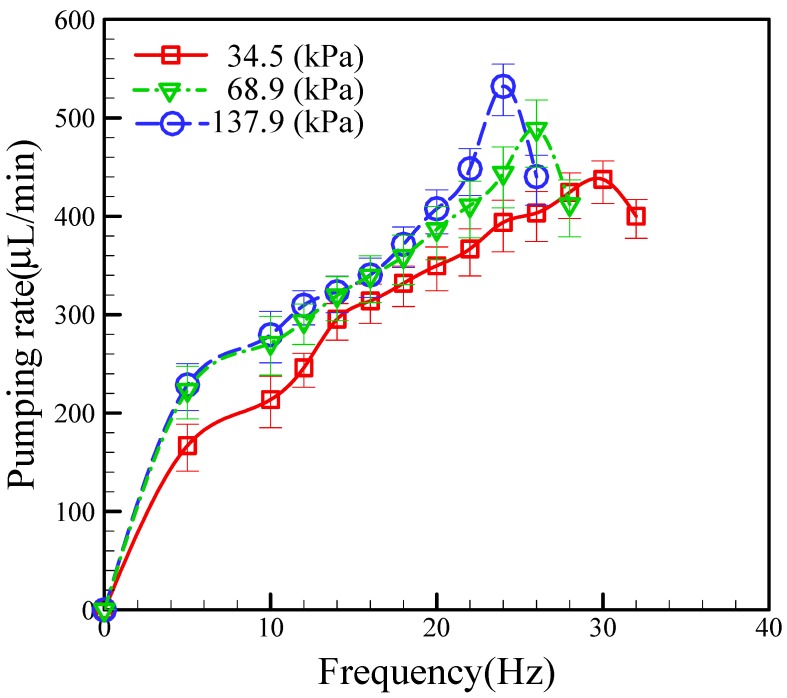
Relationship between the pumping rate and pneumatic pressures at different operating frequencies.

In this section, we fixed the location of the active check valve at 2.0 mm and investigated the effect of the applied pneumatic pressure on the pumping rate at different operating frequencies; the results are shown in Figure 7. During the test process, we sequentially varied the EMV operating frequency to examine the effect of the operating frequency. Generally, we found the pumping rate to increase with increasing the operating frequency (at a constant applied pneumatic pressure). However, the maximum pumping rate at a constant applied pressure is limited by the charge and discharge times of the compressed air. If the operating frequency is too high, the air chamber cannot be completely charged and discharged, and the pumping rate will not increase, but instead will start to fall, as shown in Figure 7. In particular, we found the optimum operational frequencies at applied pneumatic pressures of 34.5 kPa, 68.9 kPa, and 137.9 kPa to be 30.0 Hz, 26.0 Hz, and 24.0 Hz, respectively. Our experimental results also show that increased operating frequencies yield higher pumping rates at higher applied pressures. We obtained a maximum pumping rate of 532 μL/min at an operating frequency of 24.0 Hz and an applied pneumatic pressure of 137.9 kPa (see supplementary video S1). The durability test of the proposed micropump was performed to be around 10 minutes for the conditions of 137.9 kPa and 24.0 Hz. This phenomenon is reasonable since a pneumatic pressure higher than 68.9 kPa can cause the PDMS membrane to be pushed down into full contact with the glass substrate. This also explains the occurrence of the same flow rates at pressures of 68.9 kPa and 137.9 kPa.

### 4.4. Pumping Efficiency

The pumping rate is dependent on the stroke volume. Theoretically, the pumping effect increases with increasing stroke volume and, conversely, the pumping effect is reduced with decreasing the stroke volume. The stroke volume (*V_St_*) can be expressed by the following:

(3) $$V_{St}=\int_{-a/2}^{a/2}\int_{-b/2}^{b/2}\;wdxdy=\frac{2a^{2}w_{o}}{\pi^{2}}\left(\frac{\pi^{2}b}{4a}+(2-\frac{\pi^{2}}{4})\lambda \right)$$

We suppose that the mean stroke volume is related to the periodic time (*T*), as expressed by the following:

(4) $$V=\frac{1}{T}\int_{0}^{T}V_{St}\left(1-e^{-\frac{t}{T}}\right)dt=\frac{V_{St}}{e}=0.368\cdot V_{St}$$

To improve the pumping efficiency, we introduce the parameter, *η_eff_*, which is defined as follows:

(5) $$\eta_{eff}=\frac{Q_{actual}}{Q_{theo}}$$

Here, *Q_actual_* and *Q_theo_* are the experimental pumping rate and theoretical calculation flow rates, respectively. *Q_theo_* is calculated as follows:

(6) $$Q_{theo}=0.368\cdot V_{St}\cdot f_{cr}$$

Here, *f_cr_* is the experimentally critical frequency at a given applied pressure.

**Figure 8:**
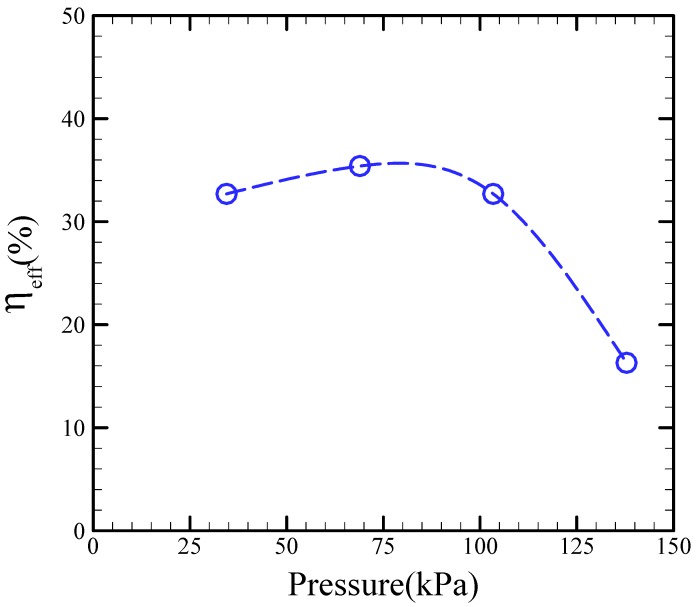
Pumping efficiency of the proposed micropump against applied pressure.

Figure 8 shows the efficiency of our proposed micropump with respect to the applied pressure. Clearly, the efficiency slightly increases with applied pressure, then attains its maximum value, and decreases with increased applied pressure. As shown in the figure, the maximum pumping efficiency is 36% at an air pressure of 68.9 kPa.

## 5. Conclusions

In this study, we proposed a new pneumatic pump that is based on a peristaltic-like actuation effect generated by the sequential deflection of an actuated membrane with an active check valve incorporated into a bottom-protruding structure. We thoroughly investigated the performance of the pneumatic pump in response to various effects. Our results indicate that the pumping rate is determined by the applied pneumatic pressure, operational frequency, and location of the active check valve. We experimentally determined the optimum air pressure, driving frequency, and location of the active check valve to be 68.9 kPa, 26.0 Hz, and 2.0 mm, respectively. We obtained a maximum pumping rate of 488.0 μL/min and a maximum efficiency of 35.4% at an air pressure of 68.9 kPa.

## Supplementary Materials

The following videos are available online at www.mdpi.com/2072-666X/9/1/1/s1, Video S1.
